# Stepping on Obstacles with a Sensory Substitution Device on the Lower Leg: Practice without Vision Is More Beneficial than Practice with Vision

**DOI:** 10.1371/journal.pone.0098801

**Published:** 2014-06-05

**Authors:** Lorena Lobo, David Travieso, Antonio Barrientos, David M. Jacobs

**Affiliations:** 1 Facultad de Psicología, Universidad Autónoma de Madrid, Madrid, Spain; 2 Centro de Automática y Robótica, Universidad Politécnica de Madrid-Consejo Superior de Investigaciones Científicas, Madrid, Spain; University of South Australia, Australia

## Abstract

Practice is essential for an adapted use of sensory substitution devices. Understanding the learning process is therefore a fundamental issue in this field of research. This study presents a novel sensory substitution device worn on the lower leg and uses the device to study learning. The device includes 32 vibrotactile actuators that each vibrate as a function of the distance to the nearest surface in a particular direction. Participants wearing the device were asked to approach an object and to step on the object. Two 144-trial practice conditions were compared in a pretest-practice-posttest design. Participants in the first condition practiced with vibrotactile stimulation while blindfolded. Participants in the second condition practiced with vibrotactile stimulation along with normal vision. Performance was relatively successful, both types of practice led to improvements in performance, and practice without vision led to a larger reduction in the number of errors than practice with vision. These results indicate that distance-based sensory substitution is promising in addition to the more traditional light-intensity-based sensory substitution and that providing appropriate sensorimotor couplings is more important than applying the stimulation to highly sensitive body parts. The observed advantage of practice without vision over practice with vision is interpreted in terms of the guidance hypothesis of feedback and learning.

## Introduction

Sensory substitution devices are devices that transform ambient energy patterns typically associated to one sense modality into patterns that can be detected through another modality. Commonly used transformations are visual to auditory and visual to tactile. Sensory substitution devices raise important fundamental scientific questions, including questions related to brain plasticity [1] and sensorimotor theories [2]. The majority of the applications of sensory substitution devices are directed to visually impaired people [3], but other applications can be found in fields such as pilot navigation, balance control, speech comprehension, and other fields [4].

Some type of training with sensory substitution devices is beneficial or even necessary [5]–[7]. Lenay and colleagues [8], for example, argued that “even the most user-friendly device will inevitably require a substantial learning process” (p. 286). These authors further claimed that the availability of appropriate learning protocols is a crucial factor for the success of sensory substitution devices. In line with such claims, the main purpose of the here-reported experiment is to contribute to the understanding of learning with sensory substitution devices. In addition to noting the importance of learning, Lenay and colleagues [8] elegantly expressed several theoretical observations that are important for the design of sensory substitution devices, some of which are related to the ecological approach to perception [9].

From the ecological point of view, perception is the picking up of higher-order variables that are useful for goal-directed behavior. To give a few examples, often-studied higher-order variables include the focus of expansion of the optic flow as specification of the direction of movement, or texture gradients as specification of terrain orientation. The ecological approach considers perception and action as two sides of the same coin; both are part of a unique process of information detection. A large number of empirical studies support the role of exploratory movements in the detection of information. Prominent among these studies are the bodies of work on dynamic touch [10] and on the concept of *exploratory procedures* [11]. Given the importance of exploratory movements in the regular functioning of perceptual and perceptual-motor systems, it seems reasonable to expect that, in order to be effective, sensory substitution systems should allow exploratory movements and sensorimotor couplings, and thereby the detection of environmental information specific to action-relevant properties.

Inspired by the ecological framework, we have previously designed and constructed sensory substitution devices that transform distance-related information into vibrotactile patterns on the torso. We experimented with these devices using tasks that are among those most typically considered by proponents of the ecological approach: the perception of obstacles [12] and of time to contact [13]. The here-presented research continues this overall approach to sensory substitution. We designed a novel device that tranforms distance-related information into vibrotactile patterns on the lower leg. An experiment is reported in which participants use the novel device to step on ground-level obstacles. The purpose of the experiment is to respond to learning-related questions.

One of the first systematic investigations of learning in sensory substitution was performed with a device referred to as the *binaural sensory aid* [14]. This device associates the distance of a target to a pitch, and the direction to an interaural amplitude difference. In the experiment reported in [14], the perception of distance and direction with the device improved after a training phase in which users received haptic feedback by touching the targets. Learning effects have also been reported in [15] and [16]. In [15], the authors used vibrotactile stimulation applied to the left index finger of participants with an *Optacon* and observed learning in the absence of feedback. In [16], a visual-to-auditory device, referred to as *the vOICe* [17], was used and visual feedback was provided without motor interaction with the environment. In addition to these and other studies with laboratory tasks, learning effects have been reported after practice with more dynamic and arguably more natural interactions with objects [18] and after the prolonged and continuous use of substitution devices outside the laboratory [19], [20].

In comparison to the large number of studies that demonstrate that learning occurs–with different devices, different tasks, and with different types of feedback as well as without feedback–few studies focus on factors that may facilitate or impair learning. Consider the following question: In learning to use a device that provides vibrotactile stimulation, what are the effects, if any, of the absence of vision during practice as compared to the possibility to rely on vision during practice? Proulx and colleagues [20] tested performance with a sensory substitution device (the vOICe) that was used during 21 days, either with or without vision. Their study, however, included only one participant in each of these conditions (as well as more participants in conditions that are not described here). Also relevant is an experiment reported in [21], in which participants learned to control a robot on the basis of tactile stimulation coupled to a camera placed on the robot. The experiment included practice phases with visual and tactile stimulation as well as practice phases with tactile stimulation only. Even so, because the purpose of the experiment was not to compare the different practice phases, all participants went through the phases in the same order, making an unbiased comparison impossible. Hence, more research is needed to understand the effects of the presence or absence of vision while learning to use non-visual sensory substitution devices.

To perform such research and to advance our broader research project, we constructed a sensory substitution device with 32 actuators on the frontal part of the lower leg. If a user stands straight up on a flat ground surface without obstacles, then all actuators vibrate with a (low) standard vibration. Deviations from this situation–which may be due to movement of the user or to the presence of an obstacle–lead to changes in the pattern of vibration. Each actuator vibrates as a function of the distance to the nearest surface in a particular sensing direction: the closer the nearest surface, the more intense the vibration. The so-computed patterns of vibration and the changes therein may allow users to perceive ground-level obstacles and to step on them. Our device does not include real sensors. Instead, to control the vibration of the actuators, the position of the lower leg is detected with movement registration cameras, and the distance to the nearest surface (either the floor or a box) is computed on-line on the basis of knowledge about the locations of the surfaces in the environment. In the reported experiment, participants wearing the device were asked to walk toward objects and to step on them.

In accordance with the issues raised above, the aims of our study are (a) to determine if it is possible to use our device to step on ground-level obstacles and, thereby, to confirm the usefulness of this type of device, (b) to determine if and how the execution of this perception-action task changes and improves with experience with the device, and (c) to determine if different practice conditions have different effects on performance. To test the effect of experience, we used a pretest-practice-posttest design with four 36-trial practice blocks. A first group of participants performed the practice blocks while blindfolded whereas a second group performed the practice blocks with vision.

Our analyses address the time needed to perform the task and several error measures: the number of trials on which the foot is lifted before reaching the obstacle, the number of trials on which the foot is not lifted sufficiently so that the obstacle is hit, and the sum of these errors. Also analyzed are the distance (from the box) at which the foot is lifted and the maximum height of the lifts. A final measure concerns exploration. Displacement by walking implies continuous changes in the tilt of the lower leg (as well as of other body segments). With our sensory substitution device the tilt of the lower leg with the device may have an exploratory function in addition to its regular function related to displacement. This is so because the pattern of vibration is a function of the structure of the environment in combination with the position and orientation of the lower leg. As an indication of this exploratory function, we computed and analyzed the range of tilt of the lower leg at a moment at which one may expect to observe exploratory movements: just before the leg was lifted to step on the obstacle. We reasoned that a more pronounced exploration should be evidenced by a larger tilt range.

## Materials and Methods

### Ethics Statement

This research project was approved by the committee for ethical research of the Universidad Autónoma de Madrid. Written informed consent was obtained from all participants.

### Participants

Twenty students and faculty members (17 women, 3 men) participated in the experiment. Their mean age was 20.2 years (*SD* = 4.3). All participants were right footed. None of them had previous experience with this sensory substitution device. In return for their participation, the participants received book vouchers at the end of the last experimental session.

### Apparatus

**Figure 1:**
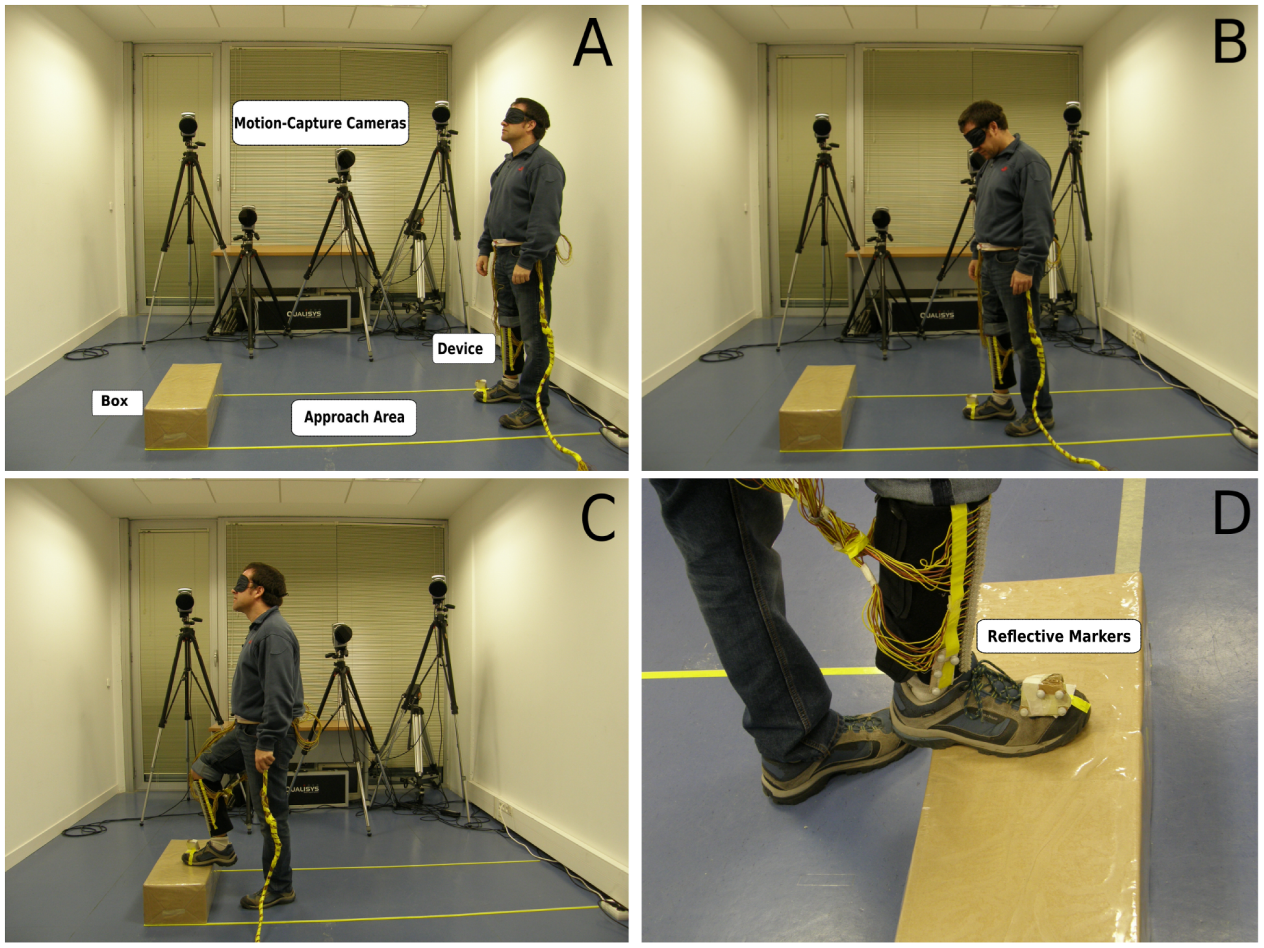
Experimental task and set-up. Participants walked through the approach area (Panels A and B) and aimed to step on the box (Panels C and D). Rigid bodies consisting of four reflective markers were attached to the right foot and to the lower right leg of the participant (Panel D). The position and orientation of these rigid bodies, and hence of the foot and the lower leg, were registered with four motion capture cameras. The experimenter was present during the execution of the task. Participants in the vision group were not blindfolded during training.

**Figure 2:**
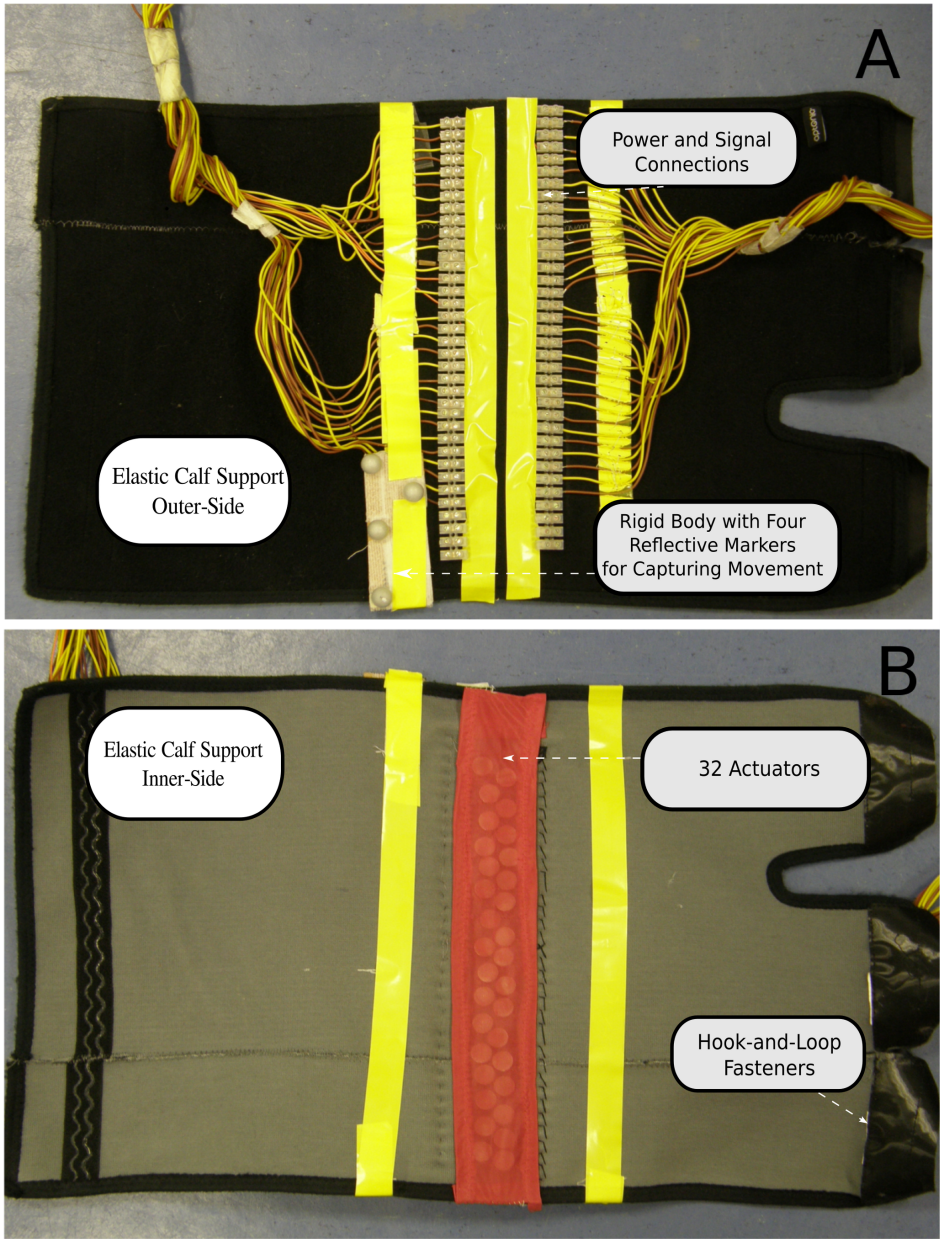
Part of the device worn on the lower right leg. The device included 32 vibrotactile actuators on the inner side of an elastic calf support. The actuators are visible in Panel B through the thin transparent fabric. A rigid body of four reflective markers was attached to the outer side of the calf support to register the position and orientation of the lower leg. Also attached to the outer side were the cables that provided power to the actuators on the inner side. The rigid body and the power cables are visible in Panel A.

Figure 1 shows the set-up and an individual (in the case of the picture one of the authors) performing the task. The set-up included an approach area of approximately 2.00×0.50 m, six cardboard boxes of different heights (0.15, 0.20, 0.25, 0.30, 0.35, and 0.40 m) placed at one of six possible distances from the participant’s starting position (1.00, 1.15, 1.30, 1.45, 1.60, and 1.75 m), and a four-camera motion capture system (Qualisys Inc., Sweden). Figure 2 shows the part of the sensory substitution device that was worn on the leg. This part consisted of 32 actuators attached to the inner side of an adjustable elastic calf support. The actuators were coin-shaped motors (6.0×3.4 mm) that were placed in a zigzag line against the tibialis anterior muscle (parallel to the shinbone). As explained in the following paragraphs, the actuators vibrated as a function of the distance to the first-encountered object in a particular direction.

The four Qualisys cameras detected the position and orientation of two rigid bodies (each formed by four reflective markers) at a frequency of 100 Hz. One of the rigid bodies was attached to the right foot and the other one to the part of the device worn on the lower leg. The position and orientation of the rigid bodies were exported from the Qualisys software to MATLAB with the MATLAB plug-in of the Qualisys software. All on-line processing was done on a single PC (Intel Core i7, 3.07 GHz). The output of the on-line processing with MATLAB was an array of 32 driving voltages. These voltages changed with the participants’ movements. The digitally-computed voltages were transformed into analog signals with two 16-channel digital/analog (D/A) conversion cards (NI-9264, National Instruments, Texas). The output of the D/A conversion cards was adjusted to the currents required by the actuators with two 16-channel printed circuit boards.

The on-line computations of the driving voltages were based on the positions and orientations of the actuators (derived from the measured position and orientation of the rigid body on the lower leg) in combination with predefined information about the environment (the position and height of the box on a particular trial). In the on-line computations, each actuator was connected to a virtual (i.e., imaginary) sensor. At each moment in time, the driving voltage of the actuator was a function of the distance to the first-encountered object in the direction of the associated virtual sensor. We first describe the details of the distance-voltage relation for a single actuator and then present illustrative examples of patterns of vibration for the array of 32 actuators.

**Figure 3:**
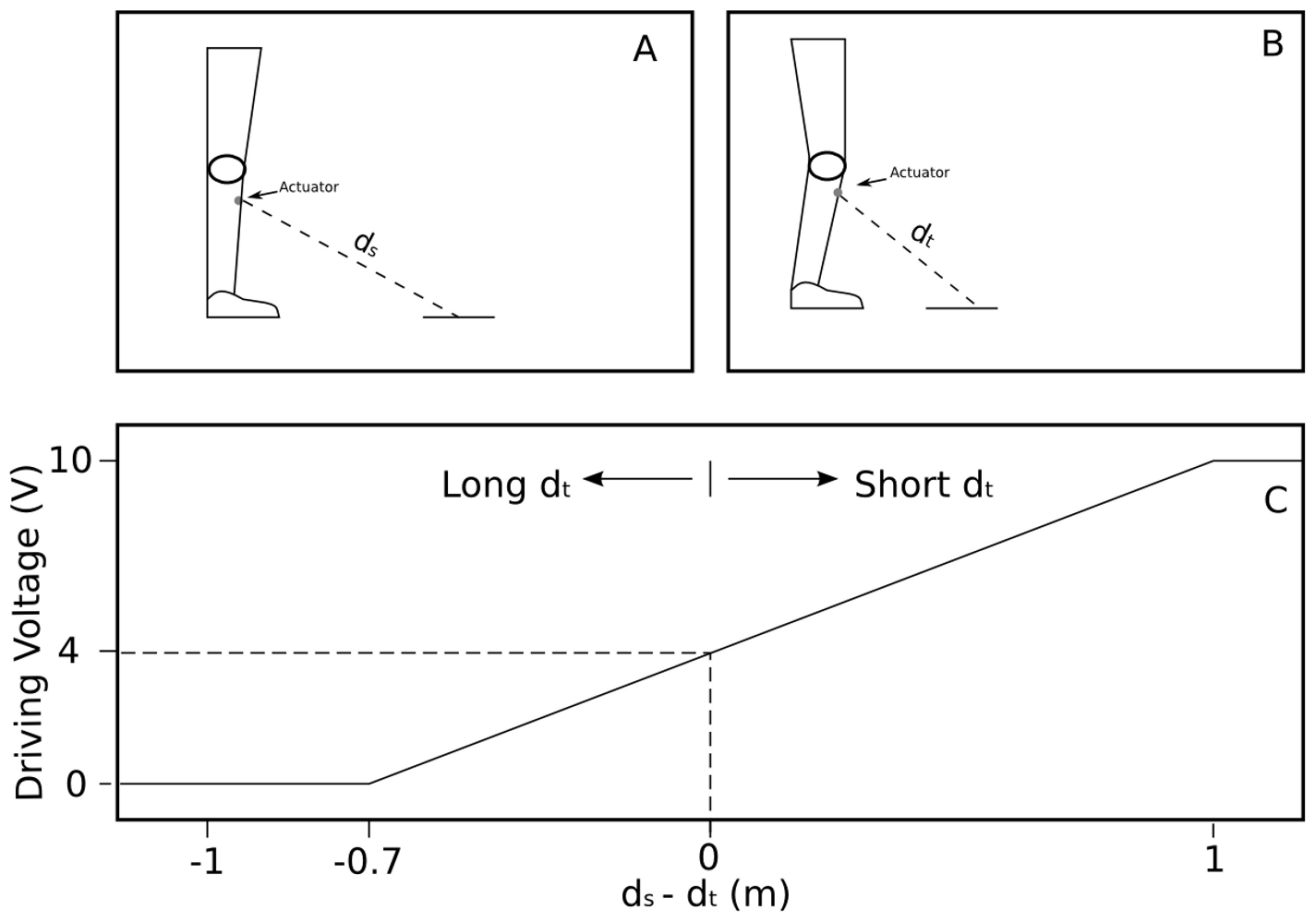
Single-actuator illustration of the distance-voltage relation. The upper left panel shows the lower leg of a participant in the standard situation with a single actuator. The dashed line indicates the direction of the virtual sensor and *d*_s_ indicates the distance between the actuator and the floor in that direction. The upper right panel shows the lower leg tilted forward, at a certain moment *t*; *d*_t_ indicates the distance between the actuator and the floor in the direction of the virtual sensor at moment *t*. The lower panel shows the digitally-computed driving voltage *v*_d_ as a function of *d*_s_ and *d*_t_: the longer *d*_t_ with respect to *d*_s_, the more negative *d*_s_–*d*_t_, and the lower *v*_d_.

The upper left panel of Figure 3 shows the lower leg with a single actuator for a participant standing straight up in an environment without box. We refer to this situation as the standard situation. The dashed line shows the direction of the virtual sensor associated to the actuator. This direction was constant with respect to the lower leg even if the lower leg moved away from the standard situation. The distance between the considered actuator and the floor in the standard situation is indicated with the actuator specific value *d*_s_ (with *d* standing for distance and *s* for standard situation). The upper right panel shows a situation in which the lower leg has been tilted forward. In this situation the distance between the actuator and the floor in the direction of the virtual sensor, indicated by *d*_t_ (with *t* indicating that this is a time-specific distance), is shorter than *d*_s_. The digital driving voltage, *v*_d_, was computed from the relation between the changing *d*_t_ and the constant *d*_s_, using the following formula: *v*_d_ = 4+6×(*d*_s_–*d*_t_). The lower panel of Figure 3 illustrates the dependence of *v*_d_ on *d*_s_–*d*_t_ defined by this formula. Note from the figure that the driving voltage of an actuator was 4 when *d*_s_–*d*_t_ = 0 (e.g., in the standard situation). The driving voltage decreased linearly until its minimum of 0 for *d*_s_–*d*_t_<0 and the driving voltage increased linearly until its maximum of 10 for *d*_s_–*d*_t_>0 (i.e., when the actual distance was larger or smaller than the one in the standard situation, respectively).

**Figure 4:**
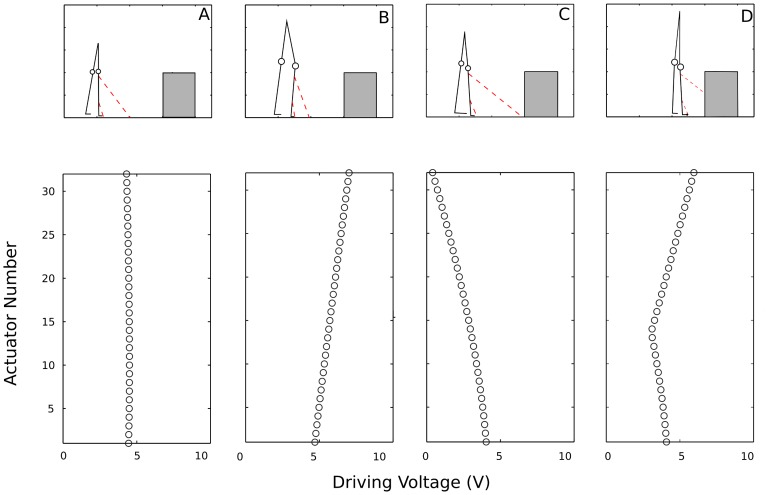
Representation of the 32 driving voltages in four common situations. The upper panels show the position and orientation of the participant’s legs (continuous lines with circles representing the knees), the sensing directions of the actuators with the highest and lowest positions on the leg (dashed lines), and the cardboard box (gray area). The lower panels show the driving voltages for all actuators associated to the situations depicted in the upper panels. The vertical axis of the lower panels gives the actuator number, with 1 being the actuator with the lowest position and 32 being the one with the highest position. Four situations are represented (from left to right): A) A participant standing straight up at a sufficiently long distance from the box (the standard situation). In this situation, the driving voltage and hence the intensity of vibration is the same for all actuators. B) A posture with a forward tilt of the lower leg. The distances to the ground are shorter and the driving voltages are higher than in the standard situation. C) A posture with a backward tilt of the lower leg. In this situation the driving voltages are lower than in the standard situation. D) Participant in front of a box. Distances to the first-encountered surfaces are reduced for the virtual sensors directed to the box. As a consequence, the corresponding actuators have higher driving voltages.

To provide further intuitions about the functioning of the device, Figure 4 shows four patterns of vibration for the array of 32 actuators. The upper part of Figure 4A shows a participant standing straight up without being influenced by the box (i.e., a participant in the standard situation). Because, in such a situation, *d*_t_ = *d*_s_ for each actuator, the driving voltage shown in the associated lower panel was 4 for each actuator. With the participant’s movements, the 32 values for *d*_s_ remained constant but the values for *d*_t_ changed, giving rise to higher driving voltages for shorter distances (Figure 4B; participant leaning forward) and lower driving voltages for longer distances (Figure 4C; participant leaning backward). The presence of a box in the scanning area also affected the vibrotactile pattern (Figure 4D).

The directions of the virtual sensors with respect to the lower leg, which were a crucial part of these computations, were determined as follows: In the standard situation, the highest actuator had its virtual sensor directed to the point on the ground 100 cm in front of the participant. Likewise, the lowest actuator had its virtual sensor directed to a point on the ground 20 cm in front of the participant. Sensors associated to in-between actuators were proportionally directed to in-between points on the floor. More details concerning a similar device and concerning the relation between the digitally-computed voltages, the analog signals, and intensity of vibration can be found in [12].

### Experimental Procedure

Initially the experimenter provided a brief explanation about the sensory substitution device and about the task: “This device includes an array of actuators that vibrate as a function of the first-encountered object on your way. If you are standing straight up, the vibration is homogeneous for all actuators. When the distance to the ground or to an object decreases, the intensity of the vibration of the actuators that are pointing to that surface increases. Conversely, when distance increases, the intensity of vibration of the corresponding actuators decreases. Your task is to walk through the approach area until you detect a box and to step on the box with your right foot. Only forward walking is allowed. A trial ends when you put your foot on the box. The distance to the box and its height will vary randomly.” After these instructions, the experimenter attached the device and the first rigid object with markers to the participant’s leg and the second rigid object to the right foot. Participants tried the device out during one preliminary trial with full vision. Participants started from the further edge of the approach area on all trials. Trials started with a “go” signal by the experimenter and finished when the participant stepped on the box, or, in case of a failure, displaced the box by kicking against it.

Participants performed three sessions of approximately one hour each on different days. During the first session participants accomplished the pretest and one practice block, during the second session two practice blocks, and during the third session one practice block and the posttest. The pretest, the four practice blocks, and the posttest each consisted of 36 trials (i.e., 36 attempts to step on the box), obtained from the factorial combination of the six above-mentioned box heights and distances. The time between the first and the third sessions was less than one week. Participants were randomly assigned to one of two groups. The vision group had full vision during the practice blocks and the no-vision group performed the practice blocks while blindfolded. All participants were blindfolded during pretest and posttest. The overall structure of the experiment is illustrated in Table 1.

**Table 1:** Distribution of the 36-trial test phases and the 36-trial practice blocks over the three 1-hour experimental sessions.

Session 1	Session 2	Session 3
Pretest (no vision)	Practice Block 2	Practice Block 4
Practice Block 1	Practice Block 3	Posttest (no vision)

**Note.** The vision group performed the 36*×*4 = 144 practice trials with vision and the no-vision group performed the practice trials without vision.

### Dependent Measures

**Figure 5:**
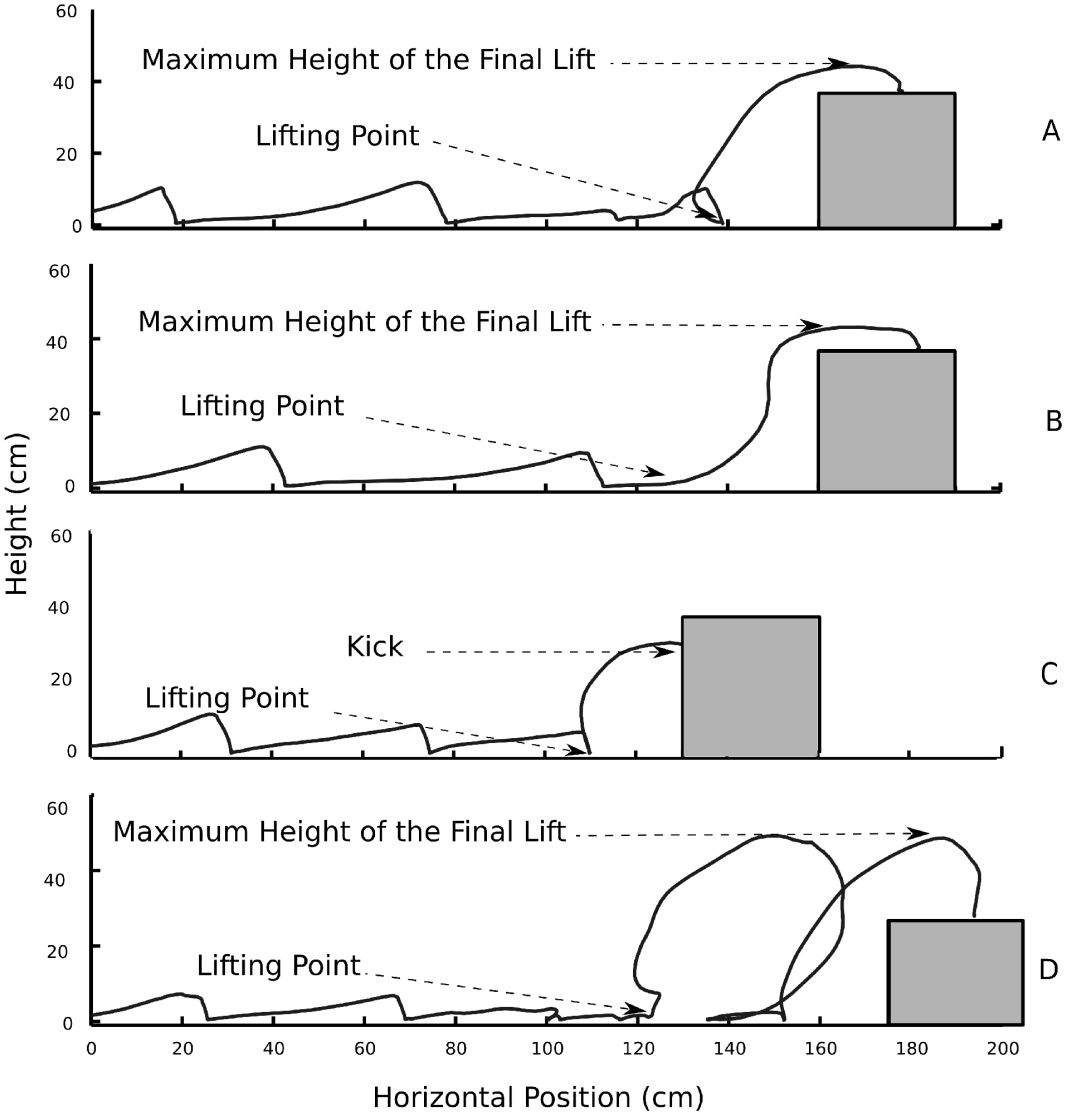
Trajectories of one participant performing four different trials. Solid black curves represent trajectories of the right foot. A) A successful trial without vision, B) a successful trial with vision, C) a trial with a kick after raising the foot, and D) a trial with a false step. As were all other trials with kicks and false steps, the trials represented in Panels C and D were performed without vision. The main points used to compute the dependent variables are identified in each of the shown trajectories.

The dependent variables listed in this subsection were obtained from the recorded movements. They were first automatically computed with MATLAB routines and then visually checked (and if necessary corrected) on a trial-by-trial basis. To facilitate the description of the variables, Figure 5 illustrates trajectories of the right foot for several representative trials.

#### Trial duration

A first dependent measure, trial duration, was defined as the time between the initiation of the movement of the right foot (speed > 20 cm/s) and the moment of the first contact of the foot with the box.

#### Kicks and false steps

Kicks, as illustrated in Figure 5C, were defined as cases in which participants contacted the vertical front surface of the box instead of the top of the box. False steps, illustrated in Figure 5D, were defined as cases in which participants lifted the foot to step on the box but in which the ground was contacted again before contacting the box, typically because the step was initiated to far from the box. Note that a strategy-dependent trade off may occur between false steps and kicks. For example, the probability of false steps is reduced at the expense of the kicks if the foot is lifted less frequently (in the extreme, not lifting the foot at all would lead to 0% false steps and 100% kicks). Because of this trade off, we analyzed the total amount of errors in addition to analyzing the kicks and false steps in isolation. The total amount of errors was defined as the sum of the kicks and false steps.

#### Distance between first lift and box

For each trial with one or more lifts of the right foot, we defined the lifting point as the initiation point of the first lift. This measure is illustrated in all panels of Figure 5.

#### Height of final lift

For trials without kicks, we determined the maximum height of the final lift, as illustrated in Panels A, B, and D of Figure 5.

#### Tilt of lower right leg

The range of tilt of the lower right leg was defined as the maximum of the forward tilt minus the minimum of the forward tilt, in degrees and with respect to the vertical, during the interval from 2 until 1 s before the first lift. This time interval was chosen because before the lift one may expect exploratory movements and because preliminary analysis showed that in the interval from 1 until 0 s before the lift the variation in the tilt was large due to the actual lifting action.

### Statistical Analysis

For each of the dependent variables listed in the previous section, we performed a 2×2 analysis of variance (ANOVA) with practice condition (vision, no vision) as between-subjects factor and test phase (pretest, posttest) as within-subjects factor.

## Results

This section first describes the overall performance, then considers the effects of practice, and, lastly, compares the effects of the practice conditions with and without vision.

### Overall Description of Performance

#### Trial duration

On average, the trial duration was 8.24 s (*SD* = 2.7). Participants in the vision group performed the training trials with vision noticeably faster than their pretest and posttest trials without vision (6.6 vs. 7.9 s; *t*(9) = 7.12, *p*<.001). This difference reached significance also for participants in the no-vision group (7.8 vs. 8.8 s; *t*(9) = 2.35, *p* = .04), who performed the practice trials as well as the pretest and posttest trials without vision.

#### Kicks and false steps

In the 36-trial pretest and posttest blocks, the mean number of errors (i.e., kicks plus false steps) was 18.8 (*SD* = 4.9). On average, participants had at least one error in 17.1 trials (*SD* = 6.9). The performance with the lowest number of errors consisted of 2 errors in a posttest (kicks in this case). The performance with the highest number of errors consisted of 35 errors in 30 trials of a pretest (30 kicks and 5 false steps). The number of kicks was larger than the number of false steps for all but one of the participants. The participant who showed a reversed pattern had 11 false steps and 6 kicks in the pretest and 10 false steps and 10 kicks in the posttest. Overall, the percentage of pretest and posttest trials without any error was 52.6%.

#### Distance between first lift and box

The average distance between the lifting point and the box was 22.2 cm in the pretest and 24.0 cm in the posttest. Arguably, however, a better detection of the distance of the box with our sensory substitution device is reflected by a lower standard deviation of the distance rather than by the average distance. This is so because in contrast to a higher or lower average distance, a lower standard deviation indicates the ability to more precisely determine the point at which to lift the foot. In the following, we therefore report analyses with the standard deviation of distance as dependent variable. Let us mention that the same analyses with average distance as the dependent variable did not yield significant results (*p*>.05).

An alternative measure for the precision of the initiation of the lift is the correlation between the position of the lift initiation and the box. On average, this correlation was 0.73. The relatively high value of this correlation indicates that the sensory substitution device provides a relatively good sensitivity to the distance of the box.

#### Height of final lift

The average height of the final lift was 42.2 cm (*SD* = 4.7). The correlation between the height of the final lift and the box was 0.29. The moderate value of this correlation indicates that participants did not show as much sensitivity for box height as they did for box distance.

**Figure 6:**
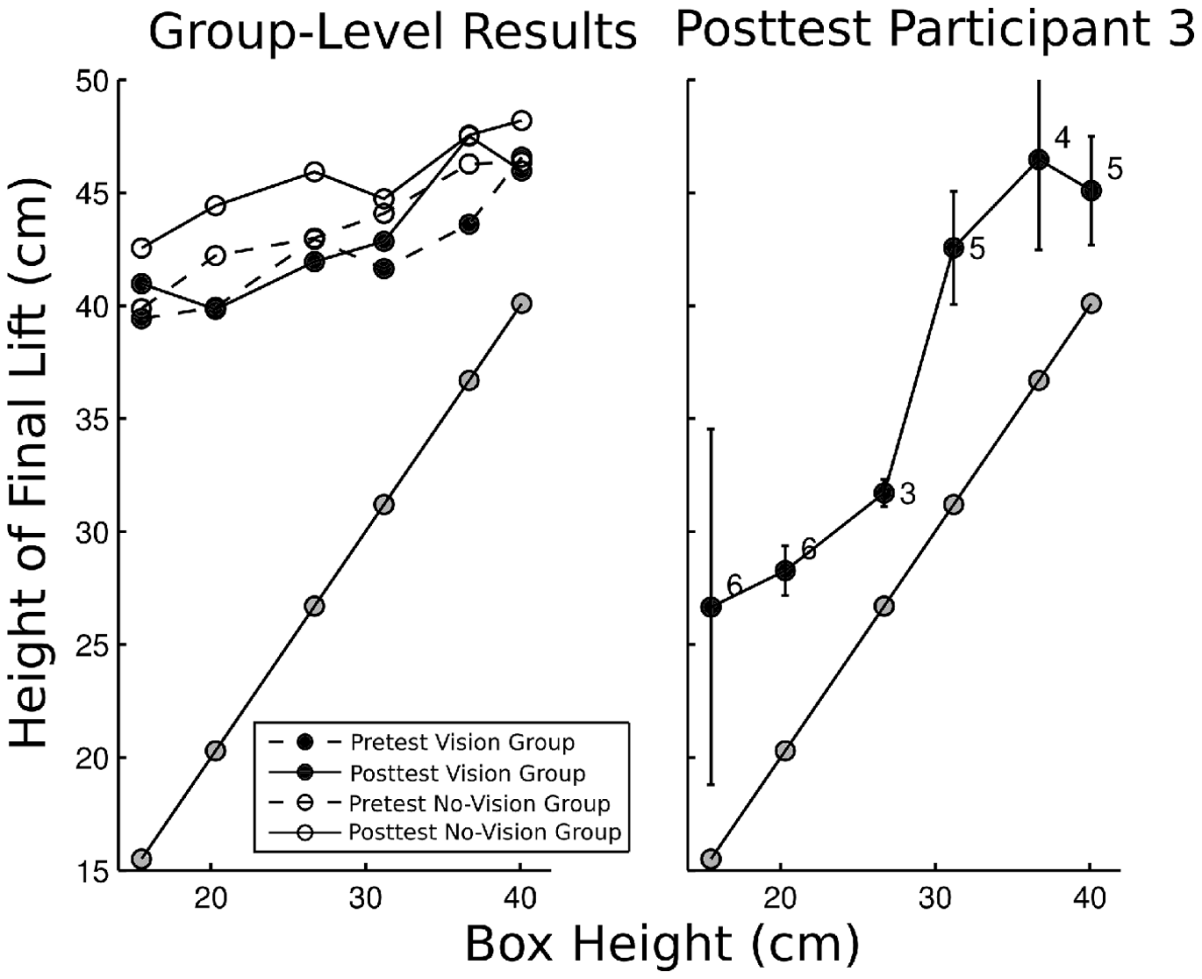
Maximum height of the final lift relative to the height of the box. Left panel: average results per group and per test phase. Right panel: posttest results of Participant 3. Error bars indicate standard deviations; numerals indicate numbers of trials used to compute the average; straight diagonal lines indicate actual box heights.

More detail is provided in Figure 6. The left panel of the figure shows the average pretest and posttest results for the two groups. The average height of the final step was only slightly lower for the low boxes than for the high boxes. Hence, rather than adjusting the final step to the height of the box, participants tended to make high steps. As long as the height of the step was higher than the highest box used in the experiment, this strategy allowed successful performance. For this reason, the results related to box height are less interesting and height-related results are not reported in the following sections.

Let us mention, however, that although the average results discard that the maximum height of the steps is strongly related to the height of the used boxes, results from individual participants occasionally indicate that it may be possible to detect box height with our device. For example, for the block of trials shown in the right panel of Figure 6, the height of the steps appeared to be adjusted to the height of the box (*r* = 0.87, *p*<.001).

#### Tilt of lower right leg

On average, 2 s before the moment of the first lift the forward tilt of the lower right leg was 7.8 deg (*SD* = 4.7) and 1 s before that moment the tilt was 5.9 deg (*SD* = 6.7). The average range of the tilt in this interval was 6.9 deg (*SD* = 5.3).

### Pretest versus Posttest and Exploration

Table 2 presents the results of the 2 (pretest, posttest)*×*2 (vision condition, no-vision condition) ANOVAs performed on the individual block averages of the previously described measures. The main effect of practice condition was never significant (all *p*s>.35), which is not surprising because at least in the pretest one does not expect to observe group differences. We now turn to the main effect of test phase. The variables that showed a significant change from pretest to posttest (*p*<.05) were trial duration, number of kicks per trial, total number of errors (kicks plus false steps) per trial, and tilt range. Trial duration decreased from 9.10 to 7.39 s, the number of kicks per trial decreased from 0.55 to 0.35, and the number of errors per trial decreased from 0.66 to 0.43. These results indicate that performance with our sensory substitution device improved with practice.

**Table 2:** Results of 2×2 Repeated-Measures ANOVAs on Dependent Variables Defined in Materials and Methods Section.

	Practice Condition		Test Phase		Interaction		
	(Vision *vs*. No Vision)		(Pretest *vs*. Posttest)				
Dependent Variable	*F*(1,18)	*p*	*F*(1,18)	*p*	*F*(1,18)	*p*	*n*
Trial Duration	0.05	.825	20.52	<.001	0.12	.732	1388
Kicks per Trial	0.87	.368	19.34	<.001	1.62	.219	1395
False Steps per Trial	0.91	.356	1.18	.200	3.89	.064	1428
Errors per Trial	0.24	.630	26.35	<.001	4.98	.039	1394
Distance of Lift to Box (*SD*)	0.69	.410	0.92	.351	6.32	.022	1407
Tilt Range	0.36	.558	7.85	.012	0.32	.578	1237

**Note.** The ANOVAs were computed on the individual block averages of the listed variables (with the exception of Distance of Lift to Box, which was performed on the *SD*s; see text for explanation). The number *n* in the rightmost column refers to the total number of valid trials used to compute the block averages (or *SD*s).

**Figure 7:**
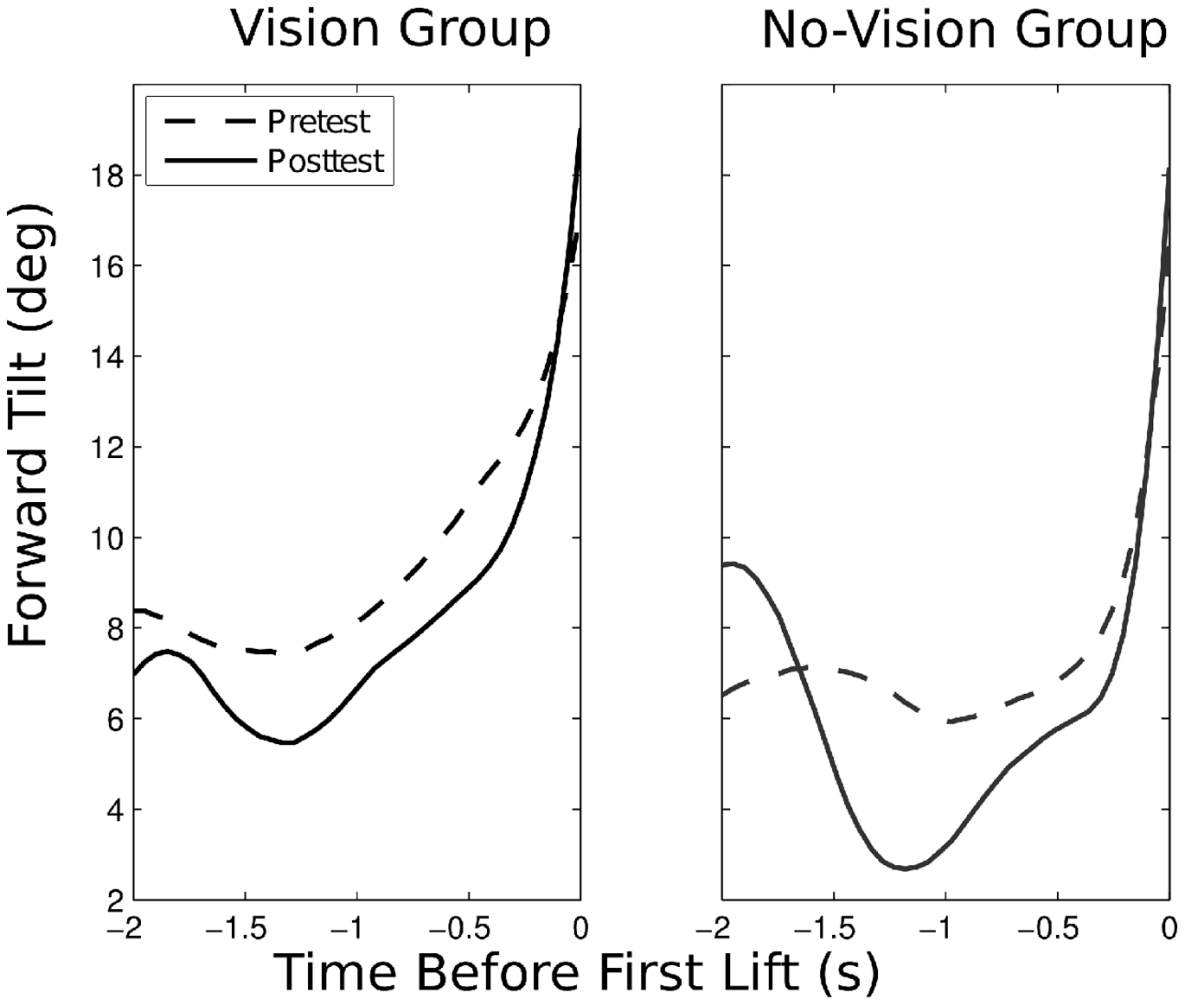
Evolution of the forward tilt of the lower right leg. Shown are the averages of the tilt angles in the final 2-vision groups. In the posttest, a decrease in the tilt can be observed between −2 and −1 s, leading to a larger tilt range in that interval.

To illustrate the significant change in tilt range, Figure 7 shows the average tilt angles in the pretest and posttest for the vision group (left panel) and the no-vision group (right panel) in the interval between 2 and 0 s before the moment of the first lift. During the last second before the moment of the lift, the angles increased to about 16 to 18 deg, indicating a forward lean at the moment of the lift. From 2 to 1 s before the moment of the lift, the average tilt angles stayed approximately constant at values of about 6 to 8 deg in the pretest (dashed curves), but they showed more interesting patterns in the posttest (continuous curves). In this interval the averaged angles showed a decrease, reaching values below 3 deg for the no-vision group. In the Discussion we will speculate that the larger change in the tilt angles observed in the posttest may evidence a more pronounced exploratory strategy.

### Practice with and without Vision

**Figure 8:**
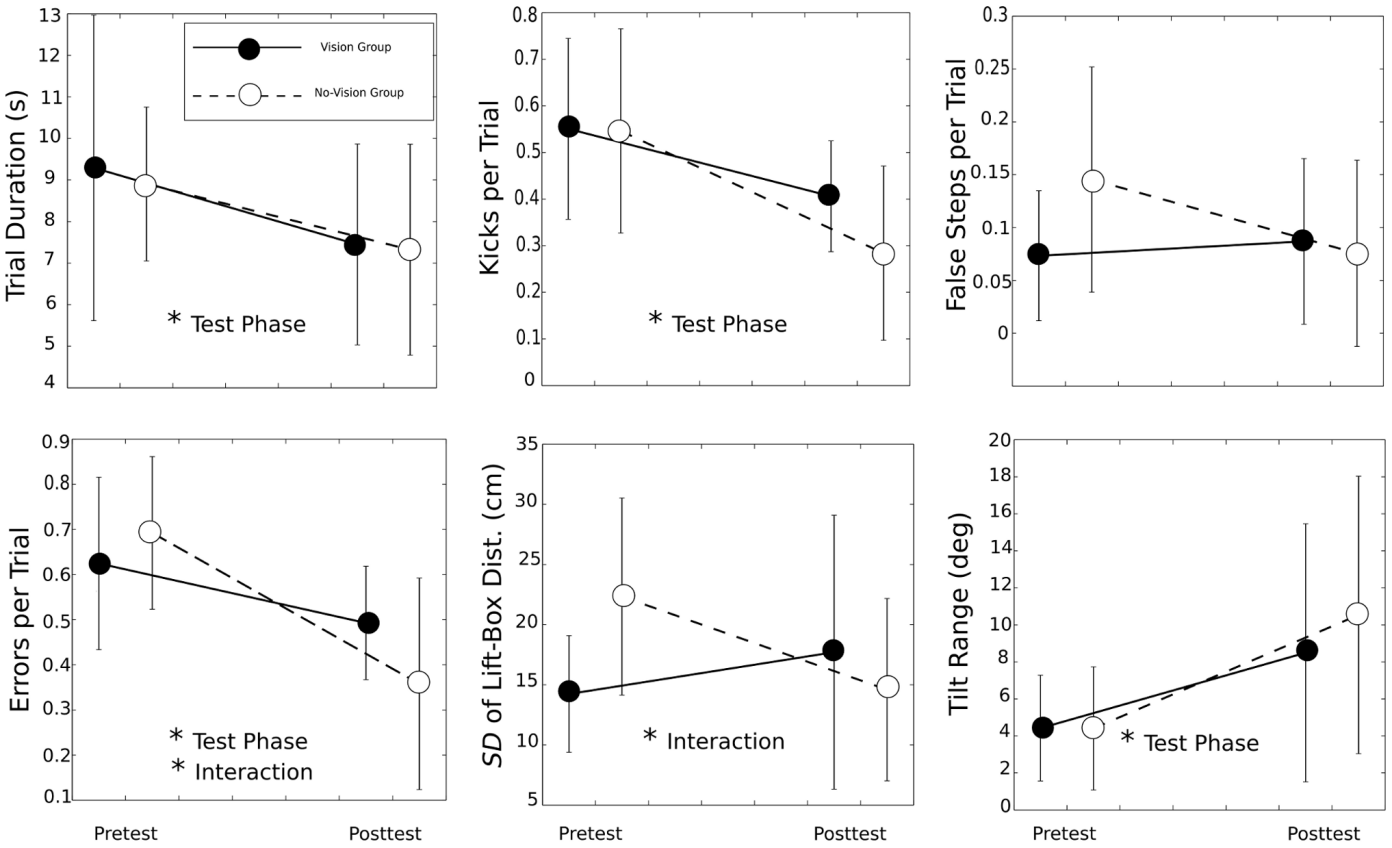
Interaction plots for the main dependent variables. Each graph shows the average value of one variable per test phase and per group. The variable names are indicated on the vertical axes. The significance levels indicated by asterisks correspond to the ones given in Table 2. Error bars represent standard deviations.

Figure 8 shows the interaction plots for the variables listed in Table 2. Results for the vision and no-vision groups are given with filled dots and open dots, respectively. The majority of the plots indicate the same tendency: Practice without vision led to a steeper improvement than practice with vision. This interaction was significant (*p*<.05) for the total number of errors and for the standard deviation of the distance between the first lift and the box. The errors per trial decreased from 0.7 in the pretest to 0.4 in the posttest for the no-vision group (pretest-posttest reduction = 0.3) and from 0.6 to 0.5 for the vision group (pretest-posttest reduction = 0.1). The standard deviation of the lift-box distance decreased from 22.3 cm in the pretest to 14.6 cm in the posttest for the no-vision group (pretest-posttest reduction = 7.7 cm) but increased from 14.2 to 17.7 cm for the vision group (pretest-posttest reduction = −3.5 cm). To summarize these results, practice without vision leads to fewer errors and to a more precise control of the moment of the first lift.

## Discussion

The aim of this research was threefold. First, we wanted to determine if it is possible to detect and step on ground-level obstacles with our sensory substitution device on the lower leg. Second, we wanted to know if performance improves with practice. Third, we tested if different practice conditions have different effects on performance. Our results indicate that these questions can be answered affirmatively.

With regard to our first aim, the average percentage of trials that were performed without errors was relatively high given the difficulty of the task (the task was difficult because the location and height of the box were varied from trial to trial). Furthermore, substantial variability was observed among participants: Whereas some participants were very successful, others were less so. In addition to the relatively high average performance, the performance of the more successful participants proves that the sensory substitution system offers enough information to complete the task. This may be interpreted as support for the construction of sensory substitution systems that are lightweight, allow a high level of mobility, and have an on-line coupling of the detected information to the novel stimulation so that users can exploit the new sensorimotor couplings [2], [8], [22].

One of the factors that may have contributed to the relatively high levels of performance is the fact that the stimulation provided by our device was computed as a function of distance. A substantial number of other devices use light intensity detected by a camera as the basis of the stimulation. Light detected by a camera shows large fluctuations due to changes in illumination and shading effects caused by moving objects. Our visual system has evolved to detect invariant patterns that specify (action-related) properties of interest from these fluctuations [9]. It is unrealistic, however, to expect that perception with sensory substitution devices can reach the sophistication of the visual system. Distances are not affected by fluctuations due to illumination and shading. We therefore believe that distance-based sensory substitution may eventually lead to more successful sensory substitution devices (cf. [12]–[14], [23], [24]). Note in this regard that experiments with light-intensity-based devices are often performed in well-controlled environments with predominantly black and white objects (e.g., [5]).

It is interesting to observe that users of our device were able to perform the task despite the poor tactile acuity of the lower leg. In this sense, the strategy that we followed in the development of the device is innovative. Most authors assume that the sensitivity of the skin is among the important criteria to choose the part of the body to place a sensory substitution device [4], [25]. Our device, in contrast, is placed on the body segment most relevant to the task at hand. Thus, rather than the sensitivity of the considered body part, what may be important is the suitability, to the task at hand, of the stimulation and of the sensorimotor contingencies provided by the device. Our results show that the design of our device is suited to the control of the final step with regard to the distance of the obstacle.

The evidence for the suitability of the device to control the step as a function of the height of the box is weaker. This may be so because our experimental task allowed a strategy that did not require the detection of information about box height: Participants frequently performed steps that were high enough even for the highest box. The fact that participants seemed to use a strategy that kept a part of the performed action constant, possibly because of the difficulty to detect the informational basis of that part of the movement, is reminiscent to a previously reported study about weight perception through dynamic touch [26]. In that study, a deafferented patient showed more reproducible wielding patterns than control subjects with intact proprioception. The constancy shown by the deafferented patient allowed her to estimate the weight of the lifted object visually. Hence, both the deafferented patient in [26] and the participants in our study discovered a way to perform an action successfully while performing a part of the action in way that does not require the typical informational basis of that part of the action–information about box height in our case and proprioceptive information in the case of [26].

With regard to our second aim, we observed that after practice the task was performed faster and with fewer errors (specifically with fewer kicks). This is consistent with a substantial number of previous studies that report effects of practice with sensory substitution devices (e.g., [14]–[16], [18]–[21]). We also observed a significant effect of practice on the variable tilt range, which indicates the amount of forward-backward tilt of the lower leg with the device (during a certain time interval before the leg is lifted to step on the box). In the pretest, participants showed relatively little variation in the tilt; in the posttest, the range of variation was larger. This pattern may highlight the role of exploration. Changes in the tilt of the leg cause changes in the orientation of the virtual sensors of the device, and, as a consequence, in the pattern of vibration on the leg. Such changing patterns may help the user to detect the environmental properties that co-determine the vibratory patterns (e.g., the presence of an obstacle). Previous studies in the field of sensory substitution that addressed the role of exploratory movements include [12] and [27].

A hypothetical change in exploratory movements with practice can be related to previous studies in the field of dynamic touch. Perceptual and perceptual-motor learning is often associated with a change in which informational variables are detected [28], [29]. The detection of particular informational variables, in turn, is associated with particular exploratory movements made to detect these variables [30], leading to the claim that performance improves because learners come to make better exploratory movements [31]. This reasoning indicates that changes in exploratory movements made with sensory substitution devices are consistent with the view that users improve because they come to detect more useful informational variables with the devices.

One may note from the lower right panel of Figure 4 that, with the current configuration of the system, the nearness of an obstacle goes together with an increased vibration of the higher actuators and with a discontinuity (in the figure at Actuator 14) of the change in vibration over the array of actuators. Our results demonstrate that such patterns, their change over time, and/or their sensorimotor coupling to exploratory actions contain information that allows the stepping action. We do not have more precise knowledge about the informational variables that are used by novices and by experts and about how these variables are detected. Achieving such knowledge would be interesting for theoretical reasons and because it may form the basis of more advanced training methods, for instance if this or a similar system is to be used as an assistive device. This is so because, if knowledge about variable use is available, then training methods can be based on the manipulation of the usefulness of the variables typically used by novices so that these graduate more quickly toward the variables typically used by experts (see [32]–[34] for applications of this methodology in other sensory domains).

With regard to our third aim, practice without vision led to a larger reduction in the number of errors and a larger increase in the precision of the initiation of the final lift than practice with vision. These findings may be related to the guidance hypothesis [35], [36]. This hypothesis holds that the more learners rely on some type feedback during practice, the more they come to depend on that feedback. Such a dependency has a detrimental effect on performance when the feedback is withdrawn. During practice with vision, our participants may have depended to a large extent on vision. As a consequence, these participants may not have learned to guide their action on the basis of the vibrotactile information as succesfully as participants that practiced without vision. In short, although vision was not found to prevent learning entirely, our results show an advantage of practice without vision and are hence consistent with the guidance hypothesis.

Let us conclude with two aspects that we consider crucial to the field of sensory substitution. First, we agree with Durette and colleagues [37] that laboratory experiments run the risk of being more of interest to scientists and designers than to users. This is so in part because laboratory studies do not always address practically relevant tasks. With the task chosen in the present study, we have aimed to make a step in a posive direction in this regard. Second, we agree with Lenay and colleagues [8] that there is a need to focus on training programs for coming to be proficient in the use of sensory substitution devices. In this sense our study shows that training without vision has advantages over training with vision.
